# Different routes to the same destination

**DOI:** 10.7554/eLife.00572

**Published:** 2013-02-19

**Authors:** Kachiko Hayashi, Akiko Iwasaki

**Affiliations:** 1 **Kachiko Hayashi** is at the Department of Immunobiology, Yale University School of Medicine, New Haven, United Stateskachiko.hayashi@yale.edu; 2 **Akiko Iwasaki** is at the Department of Immunobiology, Yale University School of Medicine, New Haven, United Statesakiko.iwasaki@yale.edu

**Keywords:** Toll-like receptors, UNC93B1, trafficking, AP-2, Mouse

## Abstract

The toll-like receptors that detect viral DNA and viral RNA in cells take different paths from the endoplasmic reticulum to the endosome.

**Related research article** Lee BL, Moon JE, Shu JH, Yuan L, Newman ZR, Schekman R, Barton, GM. 2013. UNC93B1 mediates differential trafficking of endosomal TLRs. *eLife* **2**:e00291. doi: 10.7554/eLife.00291

**Image** Keeping track of TLR9 location

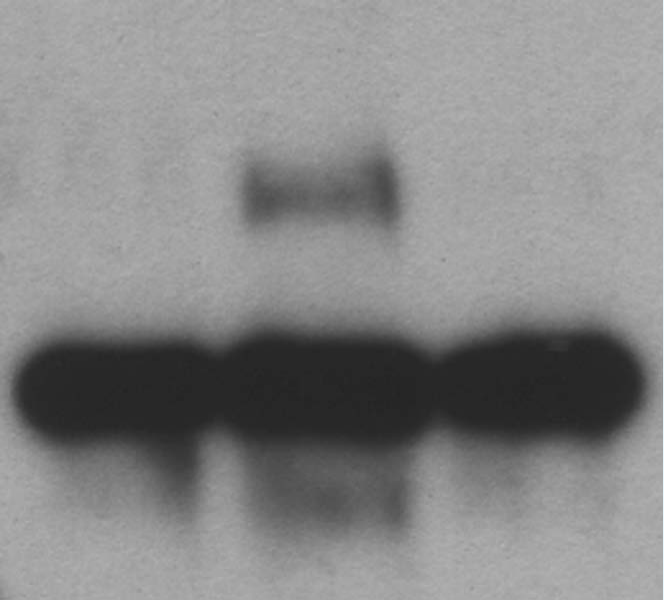

Pattern recognition receptors have two crucial roles in the innate immune system: they detect the presence of bacteria, viruses and other pathogens, and they send signals to kick-start the appropriate defence mechanisms against these microbial invaders. Over the past 15 years, toll-like receptors have emerged as an important family of pattern recognition receptors (Medzhitov, 2001). A total of 13 toll-like receptors have been discovered (including 10 that are functional in humans), with each one recognizing the distinct molecular signatures produced by a specific type of invader. Bacteria and fungi, for example, are detected via the components of their cell walls, while viruses are identified via their nucleic acids.

The toll-like receptors that detect different types of pathogens are also located in different parts of the cell and employ different signalling pathways to activate the relevant antimicrobial defence mechanisms; the receptors that detect bacteria and fungi, for example, are located at the plasma membrane, whereas those that detect viruses—such as TLR7 and TLR9—are found in compartments called endosomes. The main reason for this is the need to distinguish between the molecular signatures of the invaders and the molecules that are found naturally in the cell. The various polysaccharides that make up the cell walls of bacteria and fungi are quite different to any molecules made by eukaryotic cells, but viral nucleic acids are very similar to those found in cells. One strategy adopted by the toll-like receptors that detect viruses is to remain inactive until they are cleaved by proteases in the endosomes (Ewald et al., 2008; Park et al., 2008).

Previous studies have shown that these receptors rely on a protein called UNC93B1 to get them from the endoplasmic reticulum (where both the receptor and the protein are produced) to the endosome and to make them functional. However, it has not been clear if the complex formed by the receptor and UNC93B1 travels via the Golgi apparatus and the plasma membrane en route to the endosome (this is called the general secretory pathway; Ewald et al., 2008), or if the complex follows a more direct (but less conventional) path from the endoplasmic reticulum direct to the endosome (Kim et al., 2008). Now, in *eLife*, Gregory Barton and colleagues at the University of California at Berkeley, including Bettina Lee as first author, report that TLR9 (the receptor that detects viral DNA) reaches the endosome via a pathway that is distinct from that taken by TLR7 (the receptor that detects viral RNA), although both pass through the Golgi (Lee et al., 2013).

Previous clues that TLR7 and TLR9 are handled differently by UNC93B1 came from experiments which showed that mice harbouring a mutant form of UNC93B1 succumbed to severe autoimmune disease due to enhanced TLR7 signalling and impaired TLR9 responses (Fukui et al., 2011). The Berkeley team now shows that the UNC93B1/ TLR9 complex travels to the cell surface, where UNC93B1 recruits an adaptor protein known as AP-2 to bring TLR9 inside an endosome (route 1 in Figure 1). By contrast, a different adaptor protein, AP-4, binds to TLR7 as it leaves the Golgi to create a vesicle that travels directly to the endosomes without going to the cell surface (route 2 in Figure 1). Other endosome-based receptors (TLR3, TLR11, TLR12 and TLR13) also travel along the same AP-2 independent pathway as TLR7, but it is not known if AP-4 is required for the trafficking of these receptors.

**Figure 1.**
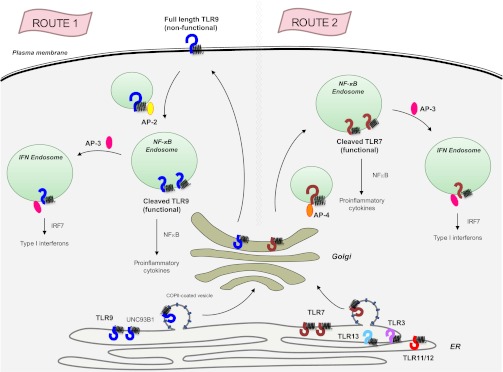
The transmembrane protein UNC93B1 (black spring-like shape) associates with various toll-like receptors (horseshoe-like shapes) in the endoplasmic reticulum to help load them into vesicles coated with COPII for transport to the Golgi. TLR9 (dark blue) follows the secretory pathway (route 1) to the plasma membrane, where AP-2 (yellow oval) binds to the UNC93B1 and transports the TLR9/UNC93B1 complex to the endosome. After being cleaved by proteases inside the endosome, TLR9 becomes active and is able to send signals to induce inflammation, which is part of the immune response. AP-3 (dark pink) can then direct TLR9 to a different type of endosome, from which it can activate the production of proteins called interferons (IFNs), which are part of the antiviral immune response. TLR7 (red) follows a different path (route 2) that does not involve going to the plasma membrane, but does result in the induction of inflammation and the production of interferons. This pathway starts with AP-4 (orange oval) binding to the receptor in the Golgi and transporting it to an endosome. Four other toll-like receptors—TLR3, TLR11, TLR12 and TLR13—also likely follow this path. AP: clathrin adaptor protein; COPII: coat protein complex II; NF-κB: nuclear factor-kappaB.

Once inside an endosome, TLR9 and TLR7 are cleaved by proteases. This allows them to detect viral DNA and RNA respectively, and to send signals that trigger inflammation, which is an important part of the immune response (Ewald et al., 2008; Park et al., 2008), and to produce interferons which, among other tasks, prevent viruses from replicating. (The adaptor protein AP-3 is required for the production of interferon; Sasai et al., 2010).

The current work adds to the mounting evidence that there is evolutionary pressure to keep the expression and function of TLR7 at a low level. First, the Berkeley team showed that translation of TLR7 mRNA into protein is inefficient, possibly due to TLR7 codon usage being highly suboptimal, and this results in levels of TLR7 that are often too low to be detected by Western blot (Lee et al., 2013). Further, the Berkeley work suggests that sending TLR7 to the plasma membrane might pose an unacceptable risk for the immune system by exposing the receptor to extracellular RNA (that was not produced by the virus). Although TLR7 has a built-in safety mechanism—it needs to be activated by a protease, a process that normally occurs in the endosome—extracellular proteases might also activate TLR7 under certain conditions.

Why must TLR7 be kept under such strict regulation? The answer likely lies in the fact that uncontrolled TLR7 signalling can cause autoimmune diseases. Gene duplication in TLR7 is sufficient, on its own, to generate an autoimmune disease that is similar to lupus (Pisitkun et al., 2006). Moreover, loss of TLR7 protects mice from lupus, while loss of TLR9 exacerbates the disease (Christensen et al., 2006). In principle, the signals sent by TLR7 and TLR9 should be very similar, so why are some autoimmune diseases associated with TLR7 but not TLR9? Lee, Barton and colleagues shed new light on this by showing that TLR7 and TLR9 associate with UNC93B1 in a mutually exclusive manner, suggesting that each UNC93B1 molecule interacts with a single receptor. This suggests the intriguing possibility that TLR7 and TLR9 endosomal compartments may be quite distinct with respect to their composition and their accessibility to different viral and endogenous ligands.

Much of the research into toll-like receptors to date has been performed on cell lines and cells taken from bone marrow and grown in vitro. The next step is to study these processes in primary immune cells, notably those cell types that are implicated in lupus (such as B cells and various types of dendritic cells). The mechanisms by which these cells detect viral nuclei acids and send signals are likely to be different to the mechanisms we already know about, so there is still plenty to do.
